# The association between triglyceride-glucose index and gallstones: NHANES 2017-2020

**DOI:** 10.3389/fendo.2024.1437711

**Published:** 2024-10-22

**Authors:** Li Gong, Shujin Fan, Zhenfei Peng, Zeyao Chen, Yuzhou Liu, Yinluan Huang, Chaofan Wang, Chunli Piao

**Affiliations:** ^1^ Department of Diabetes, Bao’an Chinese Medicine Hospital, Guangzhou University of Chinese Medicine, Shenzhen, China; ^2^ The Third School of Clinical Medicine, Southern Medical University, Guangzhou, China; ^3^ Department of Endocrinology and Metabolism, Fengxian Central Hospital Affiliated to the Southern Medical University, Shanghai, China; ^4^ Department of Endocrinology, Shenzhen Hospital (Futian), Guangzhou University of Chinese Medicine, Shenzhen, China; ^5^ Department of Endocrinology and Metabolism, The Third Affiliated Hospital of Sun Yat-sen University, Guangdong Provincial Key Laboratory of Diabetology, Guangzhou, China

**Keywords:** TyG index, insulin resistance, gallstones, cross-sectional, NHANES

## Abstract

**Objects:**

It remains unclear whether the triglyceride-glucose (TyG) index has correlations with gallstones. This study aimed to investigate the association between TyG index and gallstones.

**Methods:**

Data was obtained from the 2017-2020 National Health and Nutrition Examination Survey (NHANES). Participants who provided complete data about TyG index and gallstones were included in the analysis. Multivariable regression analysis and subgroup analysis were preformed to estimate the relationship between TyG index and gallstones. Restricted cubic splines (RSC) was employed to calculate the cut off value.

**Results:**

The TyG index was independently associated with gallstones and demonstrates a clear positive correlation (OR = 1.10; 95% CI: 1.01–1.21; p = 0.033). The threshold value is 8.98, showing a positive correlation between TyG index and gallstones when the TyG index is less than 8.98 (Log likelihood ratio P < 0.001). Subgroup analysis indicates that the correlation between TyG and gallstones is mainly observed in individuals with obesity, females, younger individuals, and those with normal blood sugar levels, with these subgroups all acting as mediators between TyG and gallstones.

**Conclusions:**

Higher TyG index was linked to a higher chance of developing gallstones. Managing insulin resistance (IR) could help reduce the risk of gallstones since the TyG index is an indicator of IR.

## Introduction

Gallstones, as a common liver and gallbladder system disease, has a global incidence rate of approximately 10%-20% and is showing an increasing trend (1). The formation of gallstones is closely related to various factors including genetics, environment, dietary habits, individual metabolic status and rapid weight loss after bariatric surgery (2–5). Insulin resistance, as a significant risk factor for metabolic diseases, has received widespread attention in recent years. Insulin resistance is not only associated with the development of cardiovascular diseases and increased risk of stroke but may also have a certain degree of correlation with the formation of gallstones (6).

Triglyceride-glucose (TyG) index is obtained by dividing the fasting triglycerides by the fasting glucose level (7). As a novel indicator for assessing insulin resistance, TyG index has been widely utilized in metabolic disease research in recent years (8). In comparison to traditional methods for evaluating insulin resistance, the TyG index is characterized by its simplicity, cost-effectiveness, and efficiency (9). It can be easily calculated using the levels of triglycerides and glucose from routine blood biochemistry tests, making it readily applicable and promotable in clinical practice. Increasingly, studies have shown a significant correlation between the TyG index and various metabolic diseases such as diabetes and cardiovascular diseases (10, 11).

In a state of insulin resistance, the body’s sensitivity to insulin decreases, leading to abnormalities in blood sugar and blood lipid metabolism. This metabolic dysfunction may further affect the composition of bile, increasing the risk of gallstone formation (12). For instance, insulin resistance may result in increased cholesterol synthesis, reduced cholesterol excretion, and decreased bile acid synthesis, all of which favor the formation of cholesterol crystals (13). On the other hand, the TyG index, as an assessment indicator of insulin resistance, may reflect the degree of metabolic abnormalities in the body. Therefore, there may be some degree of correlation between the TyG index and gallstones. However, there is now relatively little research on the relationship between the TyG index and gallstones. Therefore, this study aims to explore the correlation between the TyG index and gallstones through cross-sectional analysis, to provide new ideas and methods for the prevention and treatment of gallstones.

## Methods

### Study population

**Figure 1:**
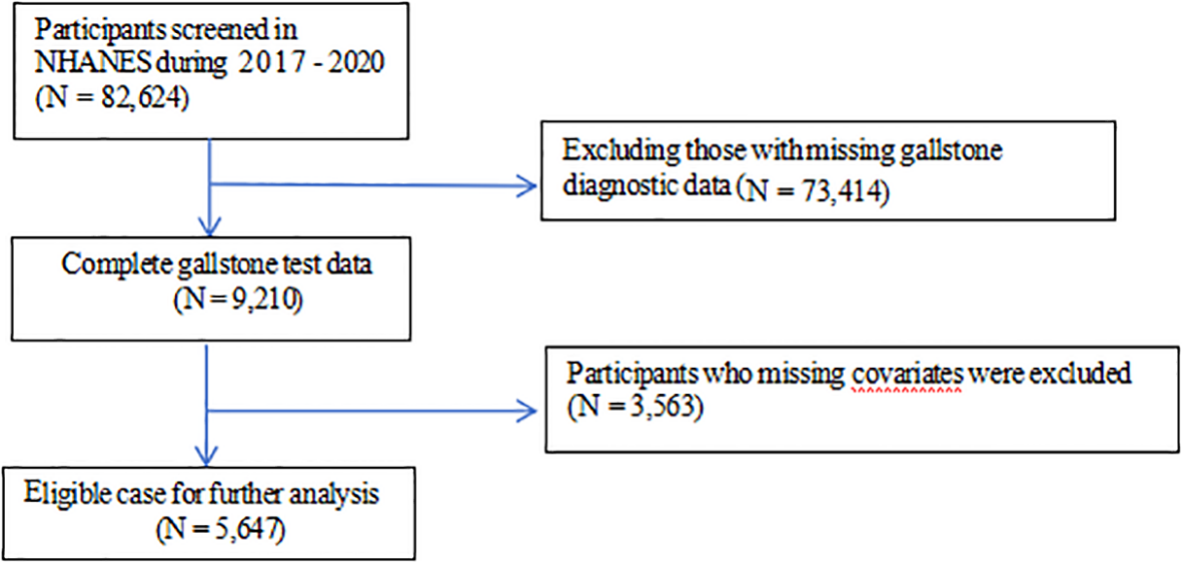
Flow chart of sample selection from NHANES 2017-2020.

The data were obtained from National Health and Nutrition Examination Survey (NHANES), a study aimed at evaluating the health and nutrition status of the US population administered by the National Center for Health Statistics (NCHS). All NHANES data are publicly available at https://www.cdc.gov/nchs/nhanes/. This cross-sectional study included 5,647 adult participants from NHANES 2017-2020. The study procedure was illustrated in detail in **Figure 1**. Exclusion criteria comprised the following: (1) participants missed data of gallstone diagnostic; (2) participants missed data of covariates. The NCHS Research Ethics Review Board granted the human subject approval for the conduction of NHANES, and written informed consent was obtained from each participants.

### Data collection

The data of TyG index was designed as an exposure variable and was calculated as Ln [triglycerides (mg/dl) * fasting glucose (mg/dl)/2] (14). Both levels of triglycerides and fasting glucose were measured by enzymatic assay using an automatic biochemistry analyzer. Concentration of triglyceride was measured using the Roche Modular P and Roche Cobas 6000 chemistry analyzers while fasting plasma glucose was measured by the hexokinase-mediated reaction using Roche/Hitachi Cobas C 501 chemistry analyzer. The prevalence of gallstones was primarily assessed by the following questionnaire rather than ultrasound examination, including “Have you ever been told you have gallstones? The occurrence of gallstones was used as an outcome variable (15). Potential covariates that could confound the association between TyG index and gallstones were adjusted in multivariate adjustment models. Covariates in our study included: age (years), gender (male/female), race categorized as Mexican American, other hispanic, non-hispanic white, non-hispanic black and other races, education level (categorized as less than high school level, high school, and more than high school level), poverty-to-income ratio (PIR), marital status (married or other), alcohol consumption (from questionnaire ALQ101-Had at least 12 alcohol drinks/1 yr? Participants who answered yes were identified as alcohol drinkers), smoking status (based on questionnaire SMQ020-Smoked at least 100 cigarettes in life, participants who answered yes were considered smokers). Hypertension and diabetes mellitus participants who answered yes were identified as having these diseases). Considering body mass index (BMI) is a risk factor for gallstones, we additionally adjusted BMI, which was found to be associated with an increased prevalence of gallstones (16).

### Statistical analysis

All statistical analyses are conducted following the guidelines of the Centers for Disease Control and Prevention (CDC) in the United States, using appropriate NHANES sampling weights, and taking into account the complex multi-stage cluster survey design in the analysis. Categorical variables were presented as a percentage and continuous variables were presented as mean ± standard deviation. We used a weighted Student’s t-test for continuous variables and a weighted chi-square test for categorical variables to evaluate the differences in groups divided by with or without gallstones. Multivariate logistic regression was employed to explore the association between TyG index and gallstones in three models. In model 1, no covariates were adjusted. Model 2 was adjusted for age, gender and race. Model 3 was adjusted for gender, age, race, poverty income ratio, body mass index, education, marital status, smoking, alcohol use, diabetes, hypertension. Restricted cubic splines (RSC) was employed to visualize the dose-response association between TyG index and gallstones after adjusting for covariates as Model 3. Subgroup analysis stratified by gender, age, married status, alcohol consumption, smoking, BMI and diabetes was also performed by stratified multivariate regression analysis. Additionally, we used the log likelihood ratio test model to test the heterogeneity of associations between the subgroups. Validate the mediating effect of the variables in different subgroups on the relationship between TyG index and gallstones using stepwise coefficient testing regression method. *P* < 0.05 was considered statistically significant. All analyses were preformed using R version 3.4.3 ( http://www.Rproject.org, The R Foundation).

## Results

### Baseline characteristics of participants

Baseline characteristics of included participants with weighted demographic were shown in **Table 1**. A total of 5,647 participants were included in this study, of whom 49.80% were female and 50.20% were male, with the average age of 50.44 ± 17.33 years. The mean level of TyG was 8.66 ± 0.66. The TyG index in patients with gallstones is higher than that in the control group, with an average of 8.784 for the gallstone patient group and 8.572 for the control group. The results of the T-test show p<0.001 for both groups. Among gallstones patients, the majority are in the age group of ≥ 60 years, predominantly female, with a BMI ≥ 30, hypertension, and a drinking habit.

**Table 1:** Baseline characteristic of participants.

Characteristic	Gallstone disease		*P*_value
	With Gallstone	Without Gallstone	
**Participants (n)**	602	5045	
**Fasting Glucose,mean(SD)**	107.8 ± 39.4	101.1 ± 36.6	<0.001
**Triglyceride,mean(SD)**	149.3 ± 96.9	138.3 ± 113.5	0.010
**TyG,median (IQR)**	8.784(0.775)	8.572(0.835)	<0.001
**Age(%)**			<0.001
18-39	1.753	29.520	
40-59	3.559	29.927	
≥60	5.348	29.892	
**Gender (%)**			<0.001
Female	7.615	42.182	
Male	3.046	47.158	
**Race (%)**			<0.001
Mexican American	1.452	10.342	
Other Hispanic	1.222	8.5	
Non-Hispanic white	4.888	34.638	
Non-Hispanic black	1.913	22.968	
Other races	1.186	12.892	
**Poverty income ratio**			<0.001
≤1.3 (%)	2.745	24.243	
>1.3 and ≤3.5 (%)	4.622	34.762	
>3.5 (%)	3.294	30.335	
**BMI (kg/m2)**			<0.001
<25 (%)	1.133	23.145	
25-30 (%)	2.993	28.281	
≥30 (%)	6.534	37.914	
**Education level (%)**			<0.001
Less than high school	1.789	13.901	
High school	2.709	21.463	
More than high school	5.398	53.976	
**Marital Status (%)**			<0.001
Married	6.552	51.921	
**Hypertension (%)**			<0.001
Yes	5.649	32.159	
No	5.012	57.181	
**Diabetes (%)**			<0.001
Yes	2.869	12.75	
No	7.792	76.589	
**Smoking (%)**			<0.001
Yes	5.525	39.986	
No	5.135	49.354	
**Alcohol Consumption(%)**			<0.001
Yes	7.367	71.365	
No	3.294	17.974	

TyG, triglyceride glucose; BMI, body mass index.

BMI was categorized as <25, 25–29.9, and ≥30 kg/m2, which corresponded to normal weight, overweight, and obese population, respectively.

### Association between TyG index and Gallstone disease

**Figure 2:**
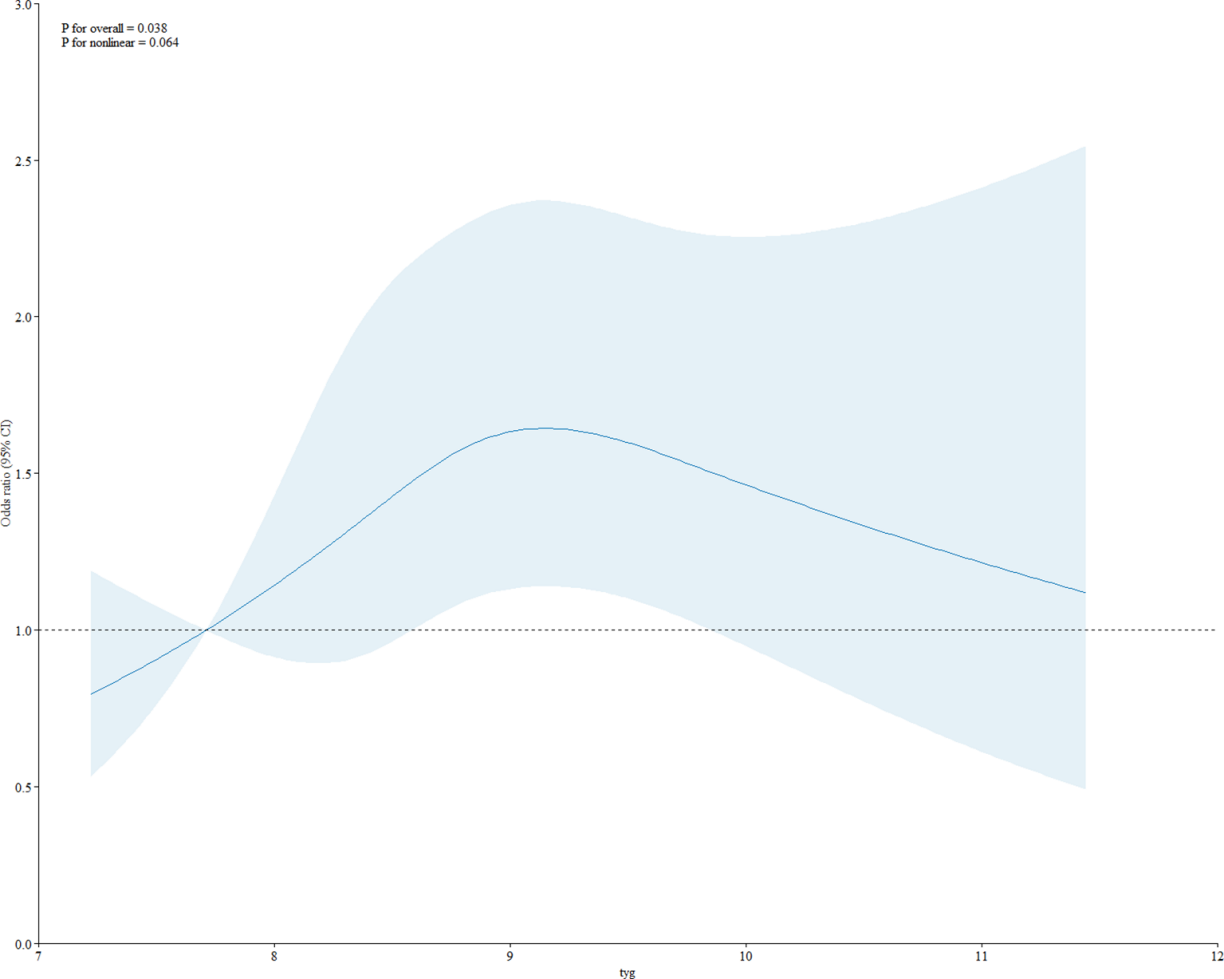
RCS of association between TyG index and gallstones.

As shown in **Table 2**, in the fully adjusted model (Model 3), a positive association between TyG index and gallstones was observed before conducting quartile analysis on the TyG index (OR = 1.10; 95% CI: 1.01–1.21; p = 0.033). indicating that each unit of increased TyG index was associated with 10% increased risk of gallstones. For conduct sensitivity analysis, we converted TyG index from a continuous variable to a categorical variable (quartiles). Compared with the lowest TyG index quartile (Q1), a significant increased likelihood of gallstones was observed in Q3 and Q4. However, the difference between Q1 and Q2 did not meet statistical significance (OR = 1.33; 95% CI: 0.99–1.78; p = 0.059). To visually demonstrate our analysis, a RSC regression line after adjusting all covariates as model 3 was performed and presented the association between serum TyG index and odds of gallstones as a U shape on a continuous scale (P for nonlinearity = 0.064), as shown in **Figure 2**. As shown in **Table 3**, we observed a non-linear relationship between TyG index and gallstones odds, identifying a cut-off value of 8.98. When the TyG index was < 8.98, a significant positive correlation with gallstone risk was observed (OR = 1.12; 95% CI: 1.01–1.24; p = 0.042). However, when the TyG index was ≥ 8.98, we did not find a significant correlation between the TyG index and gallstone (OR = 0.97; 95% CI: 0.84-1.12; p = 0.682). To quantify the strength of this relationship, we calculated the model’s log likelihood ratio, which was <0.001, confirming the statistical significance of our findings.

**Table 2:** Association of TyG with gallstone.

Exposure	Model 1		Model 2		Model 3	
	OR(95% CI)	*P*_value	OR(95% CI)	*P*_value	OR(95% CI)	*P*_value
TyG	1.31 (1.21, 1.41)	<0.001	1.29 (1.18, 1.40)	<0.001	1.10 (1.01, 1.21)	0.033
TyG quartile						
Q1, <8.19	Ref		Ref		Ref	
Q2, ≥8.19, <8.59	1.85 (1.40, 2.45)	<0.001	1.57 (1.18, 2.10)	0.002	1.33 (0.99, 1.78)	0.059
Q3, ≥8.59, <9.03	2.37 (1.81, 3.11)	<0.001	2.02 (1.52, 2.67)	<0.001	1.51 (1.13, 2.02)	0.005
Q4, ≥9.03	2.48 (1.89, 3.25)	<0.001	2.26 (1.7, 3.00)	<0.001	1.40 (1.04, 1.89)	0.029

Model 1: adjusted for no covariates.

Model 2: adjusted for age, gender, race.

Model 3: adjusted for gender, age, race, poverty income ratio, body mass index, education, marital status, smoking, alcohol use, diabetes, hypertension.

TyG, triglyceride glucose; OR, odds ratio, 95% CI, 95% confidence interval.

BMI was categorized as <25, 25–29.9, and ≥30 kg/m2, which corresponded to normal weight, overweight, and obese population, respectively.

**Table 3:** Threshold effect analysis of TyG.

	OR(95%CI)	*P_*value
TyG		
Inflection Point	8.98	
<8.98	1.12 (1.01-1.24)	0.042
≥8.98	0.97 (0.84-1.12)	0.682
Log likelihood ratio		<0.001

The results of subgroup analysis were adjusted for all covariates as model 3.

TyG, triglyceride glucose; OR, odds ratio, 95% CI, 95% confidence interval.

### Subgroup analysis

**Figure 3:**
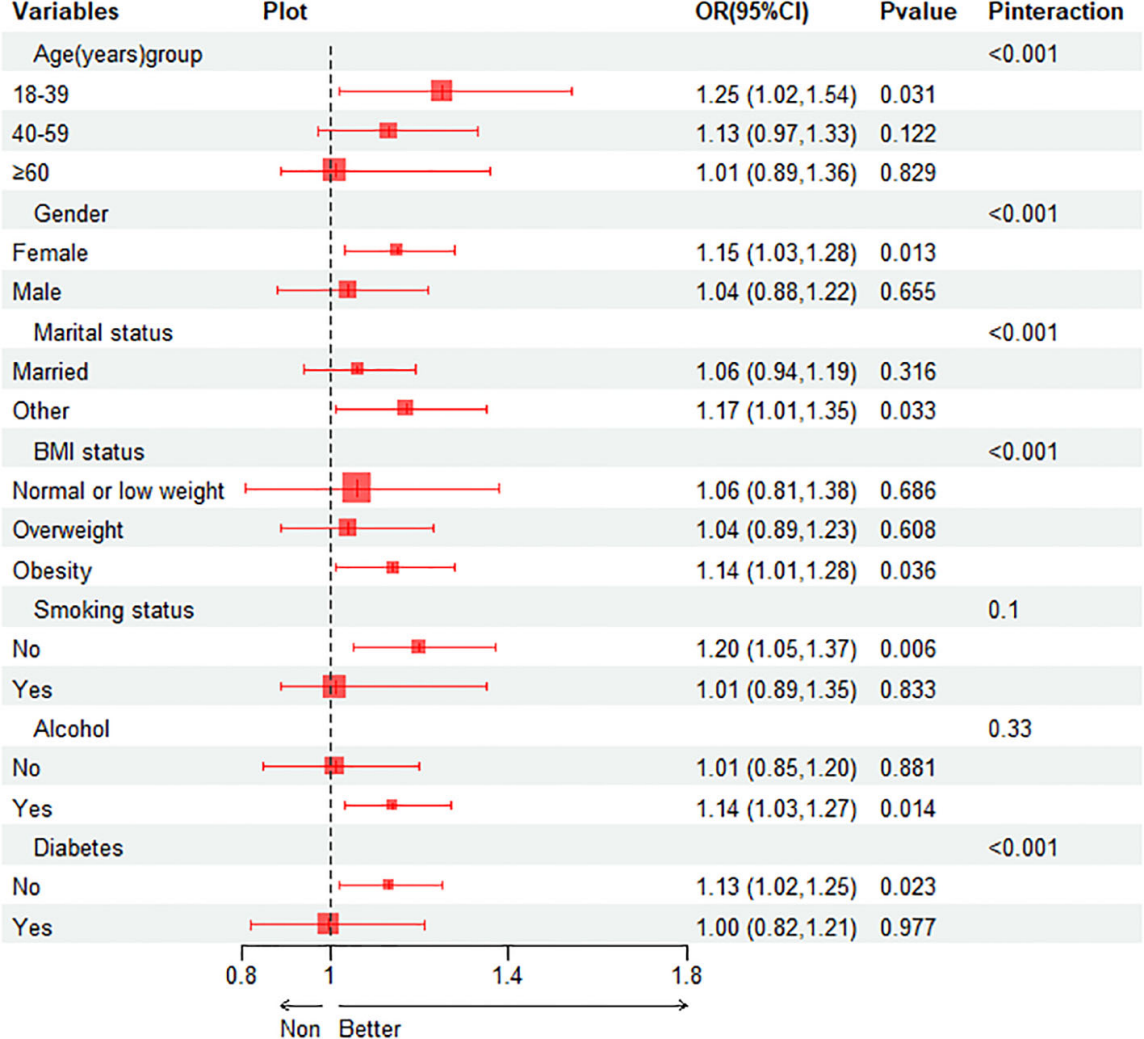
Forest map of association between TyG index and gallstones in different subgroups.

Subgroup analysis was performed to evaluate the association between TyG index and gallstones in different situations. We tested the interactions with BMI, age, gender, diabetes, smoking and alcohol. The results show that only the correlation with the p for interaction in BMI, age, gender, diabetes and married status reached statistical significance (all p for interaction < 0.001). As shown in **Figure 3**, it is evident from the subgroup analysis that participants with BMI ≥ 30, aged between 18-39, females, without diabetes and others married status exhibit a significant positive correlation between TyG index and gallstones (all P < 0.05). Subsequently, we conducted a mediation analysis on the subgroups with statistically significant P for interaction, revealing that in the influence of TyG index on gallstones, BMI accounts for 29.48% of the mediating effect, age for 17.70%, gender for 47.99%, diabetes status for 12.89% and others married status for 7.76%, as shown in **Table 4**.

**Table 4:** Subgroup and meditation analysis.

Subgroups	OR(95%CI)	*P_*value	*P* for interaction	Mediation %
**BMI**			<0.001	29.48%
<25	1.06(0.81-1.38)	0.686		
25-29	1.04(0.89-1.23)	0.608		
≥30	1.14(1.01-1.28)	0.036		
**Age**			<0.001	17.70%
18-39	1.25(1.02-1.54)	0.031		
40-59	1.13(0.97-1.33)	0.122		
≥60	1.01(0.89-1.36)	0.829		
**Gender**			<0.001	47.99%
female	1.15(1.03-1.28)	0.013		
male	1.04(0.88-1.22)	0.655		
**Married**			<0.001	7.76%
others	1.17(1.01-1.35)	0.033		
yes	1.06(0.94-1.19)	0.316		
**Diabetes**			<0.001	12.89%
no	1.13(1.02-1.25)	0.023		
yes	1.00(0.82-1.21)	0.977		
**Smoking**			0.1	
no	1.20(1.05-1.37)	0.006		
yes	1.01(0.89-1.35)	0.833		
**Alcohol**			0.33	
no	1.01(0.85-1.20)	0.881		
yes	1.14(1.03-1.27)	0.014		

The results of subgroup analysis were adjusted for all covariates as model 3 except effect modifier.

TyG, triglyceride glucose; OR, odds ratio, 95% CI, 95% confidence interval.

BMI was categorized as <25, 25–29.9, and ≥30 kg/m2, which corresponded to normal or low weight, overweight, and obesity population, respectively.

## Discussion

The TyG index is an indicator widely focused on in medical research in recent years to assess an individual’s level of insulin resistance (17, 18). Previous studies have mainly explored the correlation between the TyG index and metabolic and cardiovascular diseases (19); however, its relationship with gallstones has not been thoroughly examined. Considering that insulin resistance may lead to lipid metabolism abnormalities and dysfunction in the biliary system, thereby promoting the formation of gallstones (1), investigating the association between the TyG index and gallstones appears particularly important. Therefore, delving into the relationship between the TyG index and gallstones may not only help us to more comprehensively understand the pathogenesis of gallstones but also offer new perspectives and strategies for the prevention and treatment of gallstones.

In this study, we investigated the correlation between the TyG index and gallstones. Furthermore, we utilized subgroup analysis to explore their correlation across different populations and conducted mediation analysis on subgroups with significant differences. We found that TyG index was positively correlated with gallstones and found a breakpoint value of 8.98. Under subgroup analysis, we found TyG index in participants with BMI ≥ 30, aged between 18-39, females, without diabetes and others married status exhibit a significant positive correlation with gallstones. Additionally, the results show that BMI, age, gender, diabetes status and married status played a certain mediating role in the relationship between TyG index and gallstones.

To our knowledge, this is the first study evaluating the association between TyG index and gallstones. Previous studies have also explored the correlation between gallstones and several clinical pathological factors. In the past, the gallbladder was generally considered a simple bile reservoir organ without a metabolic regulatory role. However, an increasing amount of research has found that the gallbladder also interacts with metabolism to a certain extent. Particularly in the relationship between gallstones and insulin resistance, numerous studies have found that gallstones promote insulin resistance, and the latter is also one of the determining factors for gallstones (6). In some observational studies, insulin resistance has also been found to be a risk factor for gallstones formation, independent of body weight (20, 21). The TyG index serves as an indicator of insulin resistance, and the results of this study demonstrate consistency with previous observational research. In previous studies, HOMA-ir has been widely used as an indicator of insulin resistance to explore the relationship between insulin resistance and gallstones, without determining the range of its index correlation (22–24). In this study, the calculated TyG index cut-off value was 8.98, and there was a clear positive correlation between the TyG index and gallstones when it was below 8.98. This may suggest that in clinical practice, using the TyG index as a risk prediction value for gallstones could serve as a stratified threshold. When the TyG index is below 8.98, clinicians should be aware of the increased risk of gallstones and consider screening for gallstones, especially in high-risk patients. This may involve performing an abdominal ultrasound or referring patients for further evaluation.

Obesity has long been considered a significant risk factor for gallstones. Patients with obesity often have higher cholesterol synthesis in the liver, making it easier to form cholesterol crystals, eventually leading to the development of gallstones. Additionally, patients with obesity often have unhealthy lifestyle habits such as insufficient physical activity, which may contribute to gallbladder stasis and accumulation, further promoting the formation of gallstones. Age is also a risk factor for gallstones, with the risk of developing gallstones increasing with age. This study mainly found that the TyG index is only correlated with gallstones in the younger age group (18-39 years old). This is inconsistent with the study by Jing Wang et al., who used metabolic score for insulin resistance (METS-IR) index to investigate the relationship between insulin resistance and gallstones and found a positive correlation between the TyG index and gallstones in all age groups (25). In Ying Pan et al.’s study, explored the correlation between TyG index and various diabetes complications. It was found that in comparison to older individuals, in younger populations, the TyG index has a stronger association with ankle-brachial index and urinary microalbumin (26). In Qian Liu’s study, the role of the TyG index in indicating the risk of cardiovascular disease in postmenopausal women was explored. It was found that compared to older age groups, the TyG index is more effective at indicating the risk of cardiovascular disease in younger age groups (27). These findings suggested that when using the TyG index to explore the relationship with other diseases related to insulin resistance, perhaps the correlation could be more pronounced in younger populations. Diabetes can also increase the risk of gallstones. People with diabetes may experience an increase in fat breakdown and blood lipid disorders, leading to increased cholesterol synthesis in the liver, thereby promoting the formation of gallstones (28). Additionally, diabetes-related neuropathy can result in decreased gallbladder contraction or abnormal filling function, causing delayed gallbladder emptying and impaired bile flow, ultimately contributing to the formation of gallstones (29). In this study, we found that the TyG index did not show any correlation with gallstones in the subgroup of diabetic patients. This is inconsistent with some previous studies, such as Jingyu Su’s research, which found that the TyG index only showed a correlation with multivessel coronary artery disease in the diabetic population, but not in prediabetic or normal glucose individuals (30). In Xiang Wang’s research, an exploration of the correlation between TyG index and coronary artery disease revealed that this correlation exists only among pre-diabetic patients, not among those with normal blood sugar levels or diabetes (31). Previous studies have mainly focused on the relationship between TyG index and cardiovascular diseases in different blood glucose status populations (32). This suggests that the association of TyG index with various diseases may vary among different populations. Diabetic patients often receive medication to control their blood sugar levels. These medications, such as insulin, sulfonylureas, or metformin, not only directly affect blood sugar regulation but may also impact lipid levels (33, 34). Therefore, even in the presence of insulin resistance, these medications might indirectly influence the calculation of the TyG index by modulating blood sugar and lipid levels, thereby obscuring the true association between the TyG index and gallstones in diabetic patients. This could be one reason why there is no correlation between the TyG index and gallstones in individuals with diabetes. An increasing number of studies have found that metabolic weight loss surgery can improve metabolic syndrome and, in the early postoperative phase, can alleviate the progression of diabetes, making it a potential option for reducing the long-term incidence of gallstones (35).

Our study has several strengths. Firstly, the data we analyzed was based on NHANES data, and this study was conducted while considering the appropriate NHANES sample weights. Secondly, to ensure the reliability of our results and their applicability to a broader range of individuals, we adjusted for confounding covariates and conducted a subgroup analysis. However, there were several limitations to our study. Firstly, the diagnosis of gallstones was based on personal interview rather than ultrasound examination; the recall bias was inevitable. Secondly, due to the cross-sectional study design, we could not obtain a causal relationship between TyG index and gallstones. Thirdly, the confounding factors included in this study cannot encompass additional elements, such as factors related to blood system diseases, previous surgeries involving bypassing the duodenum, and genetic factors, all of which need to be considered in future research.

## Conclusions

Higher TyG index was associated with an increased likelihood of gallstone incidence. We speculate that using the TyG index to represent insulin resistance can to some extent indicate the risk of gallstones, especially when the TyG index is less than 8.98, which may help promote the prevention and treatment of gallstones clinically.

## Data Availability

The datasets presented in this study can be found in online repositories. The names of the repository/repositories and accession number(s) can be found in the article/supplementary material.
